# siRNA Cocktail Targeting Multiple Enterovirus 71 Genes Prevents Escape Mutants and Inhibits Viral Replication

**DOI:** 10.3390/ijms26199731

**Published:** 2025-10-06

**Authors:** Yun Ji Ga, Jung-Yong Yeh

**Affiliations:** 1 Department of Life Sciences, College of Life Sciences and Bioengineering, Incheon National University, BioComplex, Harmony-ro 265, Yeonsu-gu, Incheon 22014, Republic of Korea; dkfmal92@inu.ac.kr; 2 Center for Brain-Machine Interface, Incheon National University, BioComplex, Harmony-ro 265, Yeonsu-gu, Incheon 22014, Republic of Korea; 3 Convergence Research Center for Insect Vectors, Incheon National University, BioComplex, Harmony-ro 265, Yeonsu-gu, Incheon 22014, Republic of Korea

**Keywords:** antiviral, enterovirus, RNA interference, small interfering RNA, virus

## Abstract

RNA interference (RNAi) is a powerful mechanism of post-transcriptional gene regulation in which small interfering RNA (siRNA) is utilized to target and degrade specific RNA sequences. In this study, experiments were conducted to evaluate the efficacy of combination siRNA therapy against enterovirus 71 (EV71) and the potential of this therapy to delay or prevent the emergence of resistance in vitro. siRNAs targeting multiple genes of EV71 were designed, and the effects of a cocktail of siRNAs on viral replication were assessed compared to those of single-siRNA treatment. Cotransfection of multiple siRNAs targeting different protein-coding genes of the EV71 genome effectively suppressed escape mutants resistant to RNAi. Combination therapy with siRNAs targeting multiple viral genes successfully prevented viral escape mutations over five passages. By contrast, serial passaging with a single siRNA led to the rapid emergence of resistance, with mutations identified in the siRNA target sites. The combination of siRNAs specifically targeting different regions demonstrated an additive effect and was more effective than individual siRNAs at inhibiting EV71 replication. This study supports the effectiveness of combination therapy using siRNAs targeting multiple genes of EV71 to inhibit viral replication and prevent the emergence of resistant escape mutants. Overall, the findings identify RNAi targeting multiple viral genes as a potential strategy for therapeutic development against viral diseases and for preventing the emergence of escape mutants resistant to antiviral RNAi.

## 1. Introduction

RNA interference (RNAi) is an evolutionarily highly conserved process that controls gene expression through sequence-specific RNA degradation [1,2]. This mechanism involves the processing of double-stranded RNAs (dsRNAs) by the host RNase III-like enzyme, known as Dicer, which generates small interfering RNAs (siRNAs) of 21–23 nucleotides. The siRNA then encourages the construction of RNA-induced silencing complexes that recognize and impair homologous target messenger RNAs [3,4,5,6,7].

The rapid adjustability of nucleic acid-based therapeutics, such as RNAi using siRNAs, gives them a major advantage over other antiviral medications in responding rapidly to emerging viral infectious diseases [8]. However, even a single nucleotide mismatch within the target site can dramatically reduce the efficacy of RNAi-based viral inhibition by impairing base pairing between the guide strand and its target [8,9]. To minimize the development of resistance, researchers can use combination therapy to raise the genetic barrier, or highly potent compounds can be employed to rapidly and significantly reduce the viral replication rate, thereby reducing the formation of mutant and potentially resistant viruses [8,10].

In this study, we conducted experiments to assess the effectiveness of a combination siRNA therapy against enterovirus 71 (EV71). Our objective was to determine whether this approach of rapidly designing efficient siRNAs to target a virus could delay or even prevent the emergence of resistance in vitro. Furthermore, we aimed to identify an antiviral RNAi strategy that targets multiple viral genes, which would serve as a promising approach to therapeutic development against viral diseases and to preventing the emergence of antiviral RNAi resistance in the form of escape mutants.

## 2. Results

### 2.1. Treatment with a Combination of Multiple Sirnas Resulted in a Significant and Sustained Inhibition of Viral Replication

**Figure 1:**
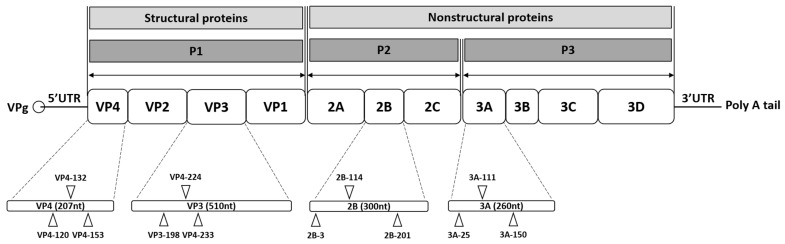
Schematic representation of the enterovirus 71 (EV71) genome and siRNA target sites. The EV71 RNA genome consists of a single open reading frame with untranslated regions (UTRs) at the 5′ and 3′ ends. At the 5’ UTR end, the genome is covalently connected to viral protein genome-linked (VPg), a small peptide that plays a crucial role in protein priming and replication. The 3’ UTR incorporates a poly-A tail. The open reading frame encodes a polyprotein that includes four structural proteins (VP4, VP2, VP3, and VP1) in the P1 region, as well as seven nonstructural proteins (2A, 2B, 2C, 3A, 3B, 3C, and 3D) in the P2 and P3 regions. Twelve target sites are indicated on the EV71 genome, corresponding to the specific siRNAs listed in Table 1. Each target site is labeled with the name of the corresponding siRNA.

In this study, the potential development of resistant viral progenies under selective siRNA pressure was investigated. Progeny viruses produced in the presence of either a single-siRNA treatment or a combination of multiple siRNAs were transferred to fresh cultures and passaged under the same siRNA pressure. The efficacy of combination treatment was evaluated by comparing HeLa cells transfected with either a single effective anti-EV71 siRNA or a combination of the four most effective siRNAs targeting VP4-132, VP3-224, 2B-114, and 3A-111 (Figure 1). Based on the evaluation of viral titers and viral protein expression, treatment with multiple siRNAs demonstrated high efficacy in inhibiting viral replication at the tested concentrations.

**Table 1:** siRNA sequences and the corresponding target gene positions in the enterovirus 71 (EV71) genome [11].

siRNA Name	Target Nucleotide Sequence (5’-3’)	GC Ratio (%)	Target Gene	Genomic Position
Scrambled control	AUUCUAUCACUAGCGUGAC	38.1	None	None
VP4-120	CUGGAAAGCAAAGUCUCA	40	VP4	865–882
VP4-132	GUCUCAAACAAGAUCCUGA	38.1	VP4	877–895
VP4-153	AGUUUGCGAACCCUGUGAA	42.86	VP4	898–916
VP3-224	CAGUUGUGUGGAUAUUACA	33.33	VP3	2019–2037
VP3-198	GUCCUUGGCAAUCCACCAU	47.62	VP3	1993–2011
VP3-233	GGAUAUUACACCCAAUGGU	38.1	VP3	2028–2046
2B-201	CAGCCACACUAGCUCUGAU	47.62	2B	3979–3997
2B-114	CUGUUGAGAAGAUCUUGAA	33.33	2B	3892–3910
2B-3	GGGUAUCUGAUUACAUCAA	33.34	2B	3781–3799
3A-150	CACCAACUAAUGUGGAACG	42.86	3A	5203–5221
3A-111	GACAGUACUGCAGGGAACA	47.62	3A	5164–5182
3A-25	CAGGCCUAUAAGAAUUAGU	33.34	3A	5078–5096

Genomic positions correspond to those of the BrCr strain, GenBank accession Nos. AB204852.1 and GU434678.1.

**Figure 2:**
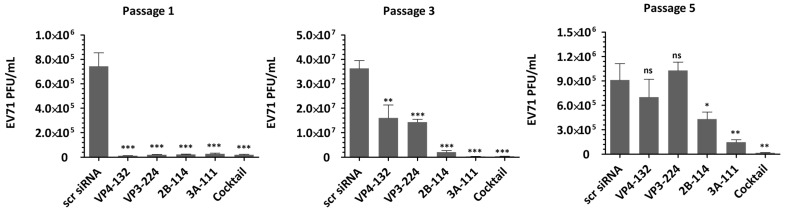
Antiviral effect of multiple-siRNA treatments. HeLa cells (2 × 10^5^ cells/well) were transiently transfected with 25 nM of individual siRNAs (VP4-132, VP3-224, 2B-114, or 3A-111) or a cocktail mixture (6.25 nM each of the four siRNAs, for a total of 25 nM) using Lipofectamine 2000 for 24 h. The cells were then infected with enterovirus 71 (EV71, strain KBPV-VR-56) at a multiplicity of infection (MOI) of 0.001. After 48 h of infection, the virus progeny present in the supernatants were repeatedly exposed to the same siRNA-transfected cells over 5 serial passages. Extracellular viral titers in the supernatant were measured by plaque assays every 48 hpi after passage 1, passage 3, and passage 5. The results are presented as a percentage relative to the scr siRNA. Data represent the mean ± SD of triplicate independent experiments. Statistical significance is indicated by asterisks denoting t test *p* values (*, *p* < 0.05; **, *p* < 0.01; ***, *p* < 0.001; and ns, not significant).

**Figure 3:**
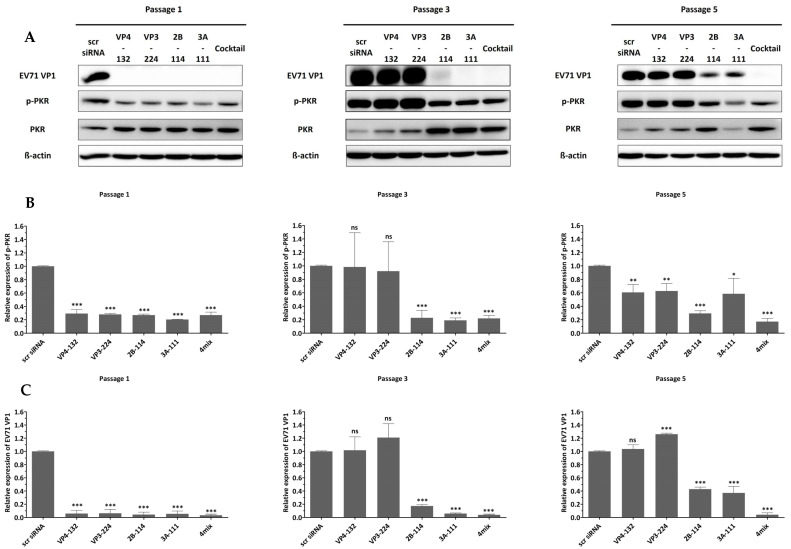
Western blot analysis of viral protein expression and PKR phosphorylation in siRNA-treated cells. HeLa cells (2 × 10^5^ cells/well) were transfected with 25 nM of siRNAs for 24 h, then infected with EV71 (MOI = 0.001) for 24 h. (**A**) Cell lysates collected at 24 hpi were analyzed by Western blot to assess the relative protein quantities of EV71-specific siRNA-treated cells compared to scrambled siRNA-treated cells (scr siRNA). β-Actin was detected as the internal control. (**B**,**C**) The intensity of each band was quantified using Image J software. The VP1 protein expression levels were normalized by β-Actin (**B**). The phospho-PKR protein expression levels were normalized by PKR (**C**). All data are presented as means ± SDs from three independent experiments, compared with the scr siRNA group. Statistical significance is indicated by asterisks, denoting t test *p* values (*, *p* < 0.05; **, *p* < 0.01; ***, *p* < 0.001; and ns, not significant).

As shown in Figure 2, treatment with individual siRNAs at a concentration of 25 nM resulted in extracellular viral titers of (1.13 ± 0.12) × 10^4^, (1.90 ± 0.28) × 10^4^, (2.20 ± 0.33) × 10^4^, and (1.97 ± 0.29) × 10^4^ for VP4-132, VP3-224, 2B-114, and 3A-111, respectively, at passage 1. At passage 3, the corresponding viral titers increased to (1.70 ± 0.43) × 10^7^, (1.44 ± 0.09) × 10^7^, (2.07 ± 0.49) × 10^6^, and (2.90 ± 0.24) × 10^5^, respectively. Among the cells treated with a single siRNA at passage 5, only the 2B-114- or 3A-111-treated cells maintained reductions of (4.30 ± 0.71) × 10^5^ and (1.47 ± 0.25) × 10^5^, respectively, in the viral titers. In contrast, the cells treated with VP4-132 and VP3-224 showed complete restoration of the reductions in the viral titers. Treatment with a combination of multiple siRNAs at a total concentration of 25 nM resulted in a significant and sustained reduction in viral titers. At passage 3, there was a decrease greater than (3.77 ± 0.37) × 10^5^, and at passage 5, a decrease of (1.63 ± 0.41) × 10^4^ was observed. However, as the pressure of single-siRNA treatment increased, the effectiveness of siRNAs against EV71 diminished, resulting in a level of VP1 protein similar to that of the scrambled siRNA group (scr siRNA), as shown in Figure 3.

To confirm that the antiviral effects were specifically mediated by siRNA, we examined PKR phosphorylation as an indicator of interferon pathway activation. Figure 3 shows that cells transfected with different siRNAs, exhibiting various inhibitory effects on EV71, did not show a significant change in phosphorylated PKR. These results indicate that the siRNAs did not induce an interferon response, and the inhibition of viral replication was solely mediated by siRNA.

### 2.2. The Cells Treated with a Combination of Multiple Sirnas Exhibited Stable Cell Viability

**Figure 4:**
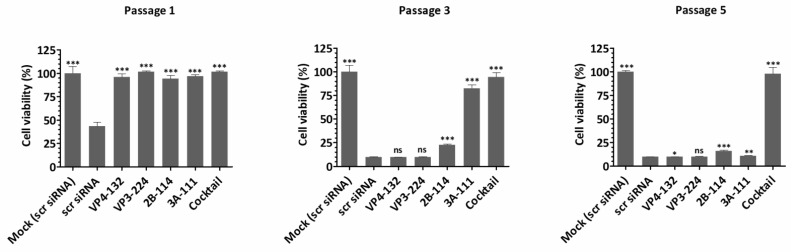
Cell viability assessment following siRNA treatment and viral infection. HeLa cells (1 × 10^4^ cells/well in 96-well plates) were transfected with 25 nM of siRNAs for 24 h, then infected with EV-A71 (MOI = 0.001). Cell survival was measured by the water-soluble tetrazolium salt assay at 48 h post infection. The results are presented as the percentage of viable cells compared to scr siRNA-treated (mock-infected) cells and as the means ± SDs of triplicate independent experiments. Virus-free cells treated with scr siRNA are represented by the scr siRNA group. Statistical significance is indicated by asterisks, denoting t test *p* values (*, *p* < 0.05; **, *p* < 0.01; ***, *p* < 0.001; and ns, not significant).

Cell viability, which represents the direct cytopathic effect of the virus, showed an inverse relationship with extracellular viral titers and viral protein expression (Figure 4). For the single-siRNA treatment, with the siRNAs VP4-132, VP3-224, and 2B-114, the cell viabilities at passage 1 were 95.99 ± 3.63%, 101.70 ± 0.64%, and 94.13 ± 3.74%, respectively. However, after passage 3, the cell viability rapidly decreased, similarly to that of the scr siRNA-treated cells. The cells treated with the single siRNA 3A-111 maintained a cell viability of 82.69 ± 3.64% at passage 3 but showed a significant decrease to 10.90 ± 0.30% at passage 5. In contrast, the cells co-treated with the four siRNAs exhibited stable cell viability, measuring 101.47 ± 1.13% at passage 1, 94.53 ± 4.57% at passage 3, and 97.66 ± 7.20% at passage 5. Therefore, combination treatment with multiple siRNAs was highly effective, resulting in a greater reduction in the percentage of infected cells compared to treatment with a single siRNA.

### 2.3. Combination Therapy with Sirnas Targeting Multiple Viral Genes Successfully Prevented Viral Escape Mutations over Five Passages

**Figure 5:**
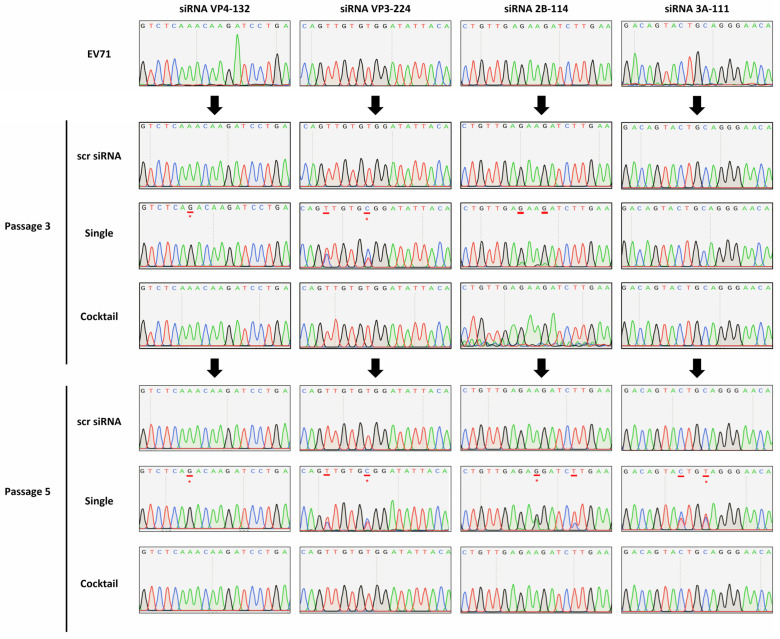
Analysis of four distinct siRNA (VP4-132, VP3-224, 2B-114, and 3A-111) target sequences in the virus collected from supernatants 48 h after infection in the indicated passages. The newly arising mutation compared to the preculture EV71 sequence (BrCr strain) is underlined, and red asterisks represent the predominant mutational positions.

The serial passaging of HeLa cells treated with a single siRNA led to the emergence of single-nucleotide polymorphisms within the targeted siRNA sites (Figure 5). At passage 3, mutations were detected for the siRNAs VP4-132, VP3-224, and 2B-114 in the single-siRNA-treated HeLa cells. At passage 5, nucleotide polymorphisms were observed in all single-siRNA treatment groups, whereas no mutations were detected in the viruses passaged through HeLa cells treated with the siRNA cocktail targeting multiple viral genes.

To be more specific, when the VP4-132 and VP3-224 groups were treated with a single siRNA, resistant viral mutants carrying A to G mutations at position 8 and T to C mutations at position 9, respectively, rapidly emerged after three passages. Additionally, at passage 5, A to G mutations at position 10 and C to T mutations at position 11 were observed for the 2B-114 and 3A-111 single-siRNA treatment groups, respectively. However, when the four effective siRNAs were combined, significant antiviral activity was maintained, and no mutations had occurred at the target site by passage 5.

Based on the inhibition of viral protein expression, the identified mutations conferred either a fully or partially resistant phenotype. The acquisition of a mutation at the target site after treatment with a single siRNA was associated with the nearly complete loss of inhibition. For siRNA VP4-132, an A-to-G transition at position seven in the VP4 target site was identified at passage 3, and this mutation persisted until passage 5 (Figure 5). In the case of siRNA VP3-224, a T-to-C transition at position nine in the VP3 target site was detected at passage 3 and remained until passage 5. However, at passage 5, the electropherogram displayed two almost equal peaks, indicating the presence of a subpopulation of viruses with this mutation. Additionally, also at passage 5, another possible T-to-C transition was observed in the VP3 target site, represented by a small C peak, suggesting the retention of this mutation in a subset of viruses.

Regarding siRNA 2B-114, although a partially resistant phenotype was observed due to the early emergence of low-level resistance, clear mutations were not identified in the 2B target regions at passage 3. However, analysis of the electropherogram at this site during passage 3 revealed the presence of small adenosine peaks alongside guanine peaks at positions eight and eleven in the 2B target region, indicating that a subpopulation of viruses had acquired this mutation between the first and third passages. By passage 5, at the 2B target region, positions eight and eleven had reverted to the G mutation, and a second novel A-to-G transition was identified at position ten. Furthermore, an additional possible T-to-C transition at position fifteen was found, although the electropherogram at this latter site displayed smaller cytosine peaks. In the case of siRNA 3A-111, viruses passaged through single-siRNA-treated cells remained susceptible to RNAi-mediated inhibition for the first three passages. However, at passage 5, a C-to-T transition at position eight was identified, and an additional possible C-to-T transition at position ten was also found, with the electropherogram showing two almost equal peaks, which suggests the presence of a subpopulation of viruses retaining these mutations.

In contrast, viruses that were passaged through a combination of multiple-siRNA-treated cells remained susceptible to RNAi-mediated inhibition throughout all five passages. Notably, mutations were not observed by passage 5 for EV1 passaged through a combination of multiple-siRNA-treated cells, indicating that the emergence of viral escape mutants from siRNA-mediated inhibition was completely prevented until passage 5.

## 3. Discussion

Despite the advantages of siRNA-based antiviral approaches, the emergence of mutations resistant to siRNA treatments remains a challenge [8]. The use of siRNA as an antiviral agent can exert selective pressure on the siRNA target, potentially resulting in the appearance of escape variants due to changes in the target sequence [12]. In particular, RNA viruses evolve rapidly under hostile conditions such as antiviral pressure. In an attempt to address this issue, siRNAs have been designed to target highly conserved viral sequences, but the virus eventually evades siRNA through evolution within a few replication cycles [9].

Unlike DNA viruses, most RNA viruses replicate using RNA-dependent polymerases without proofreading activity, leading to more frequent mutations and faster evolution under similar environmental conditions [13].

Numerous studies have demonstrated that RNA interference (RNAi) strategies can effectively inhibit EV71 replication by targeting diverse viral gene regions, both in vitro [11,14,15,16,17,18] and in vivo [19]. However, these previous single-target approaches consistently faced the challenge of viral escape mutations, particularly evident in serial passage studies where antiviral efficacy progressively declined [17]. The effectiveness of combination therapy using siRNAs targeting multiple independent regions, in terms of inhibiting EV71 replication in vitro, was evident in this study. Combination therapy with siRNAs targeting multiple viral genes successfully prevented viral escape mutations over five passages. While the cocktail showed improved efficacy, synergy modeling was not performed and remains a future objective.

The findings of this study revealed the rapid emergence of resistance following siRNA monotherapy, where a population of resistant viruses was identified after a single passage in cells treated with siRNAs targeting viral genes. To highlight this difference, we extended our investigation to five passages, by which point the antiviral effect of single siRNAs had already deteriorated, underscoring the sustained efficacy of the cocktail strategy. Although longer-term monitoring will be required to fully determine whether such mutations may revert in the absence of selective pressure, the present study demonstrated that combination therapy using siRNAs targeting multiple viral genes can effectively suppress the emergence of resistant escape mutants from RNAi-based antiviral interventions.

Despite targeting conserved regions, treatment with a single siRNA resulted in the generation of mutations in all target sites. Given the limitations of Sanger sequencing, future studies employing deep sequencing will be critical to uncovering sub-consensus escape variants and clarifying their association with siRNA-targeted sites. A single transition mutation within the siRNA complementary region of the target RNA significantly reduced RNAi efficiency. Viruses passaged through cells treated with a single siRNA rapidly developed resistance, and mutations were identified in the target sites. Consistent with previous studies [9,20,21], EV71 grown under the same siRNA conditions showed complete resistance to the same siRNA during subsequent infection cycles over three passages, similarly to the pattern of mutations observed in EV1 passaged under siRNA treatment. As Phillip McDonagh mentioned in a previous study, the loss of antiviral effect observed in this study could be attributed to the emergence of resistant isolates or the overwhelming of the RNAi machinery as cells are exposed to a progressively higher multiplicity of infection (MOI) under viral replication [21].

Target-site mutations were detected after five passages with single-siRNA treatments; however, no additional phenotypic changes, such as altered viral protein expression or increased pathogenicity, were observed. These mutations were primarily point substitutions that reduced RNAi efficiency rather than directly enhancing the replicative capacity of the virus. Nevertheless, longer-term passages or in vivo studies would be valuable to determine whether resistant variants may eventually acquire compensatory changes that influence viral replication or pathogenicity, and such analyses warrant further investigation.

The in vitro effectiveness of combination therapy in inhibiting viral replication by using siRNAs targeting multiple independent regions was evident in this study. The combination approach with siRNAs targeting different regions, including structural (VP4 and VP3) and nonstructural proteins (2B and 3A), delayed the occurrence of escape mutations. Combination therapy with siRNAs targeting multiple viral genes successfully prevented viral escape mutations over five passages. The combination of siRNAs specifically targeting different regions demonstrated an additive effect and was more effective at inhibiting EV71 replication than individual siRNAs, even at later timepoints.

The lack of effective in vivo inhibition of virus proliferation by siRNAs remains a major limitation of this study. While it was limited to HeLa cells, which are widely used for their consistent transfection efficiency and permissiveness to EV71 replication, we acknowledge that further validation in disease-relevant human cell types, such as RD, SK-N-SH, or iPSC-derived neural cells, would enhance the translational relevance of our findings. In parallel, mutagenesis-based studies will be essential to determine whether the identified substitutions directly confer resistance to individual siRNAs or cross-resistance to the siRNA cocktail. Furthermore, future hypothesis-driven studies should incorporate robust statistical frameworks such as predefined primary endpoints and appropriate multiplicity corrections to enhance their translational relevance and reproducibility across complex experimental settings.

However, the abundance of antiviral studies utilizing siRNAs built upon these findings can enhance the feasibility of designing effective siRNAs with high efficiency and specificity, particularly in unexpected pandemic situations or when variant strains with unknown genomic sequences emerge.

## 4. Materials and Methods

### 4.1. Cell Cultures and Viruses

To evaluate the antiviral activity of the siRNAs targeting multiple genes, we employed a specific EV71 strain (KBPV-VR-56). The virus was maintained in HeLa cells (CCL-2™, ATCC, Rockville, MD, USA), which were grown in Dulbecco’s modified Eagle’s medium (DMEM; Invitrogen, Carlsbad, CA, USA) supplemented with 10% fetal bovine serum (FBS; Merck Millipore, Darmstadt, Germany) and 100 μg/ml penicillin–streptomycin, under standard culture conditions with 5% CO_2_. Mock-infected cells served as uninfected controls throughout all of the experiments.

### 4.2. RT–PCR and Viral Genome Sequencing

Viral RNA was extracted using the QIAamp Viral RNA Kit (Qiagen, Hilden, Germany) and reverse-transcribed with the iScript cDNA Synthesis Kit (Bio-Rad Laboratories, Hercules, CA, USA). PCR amplification was conducted with the AccuPower HotStart Pfu PCR PreMix (Bioneer, Daejeon, Republic of Korea). For RT–PCR and sequencing, diagnostic primers [22], designed from the conserved regions of EV71 genotype A were employed, including those targeting the 5′ UTR, VP4, VP3, 2B, and 3A genes [11]. Purified PCR products were submitted to Cosmogenetech, Inc. (Seoul, Republic of Korea) for sequencing.

### 4.3. siRNA Design

Double-stranded siRNAs targeting multiple EV71 genes were designed using the Turbo si-Designer algorithm (Bioneer, Daejeon, Republic of Korea). The algorithm applies criteria such as nucleotide composition, consecutive base repetition, thermodynamic stability, energy profile, and base preference to optimize siRNA efficacy [5,23,24,25]. Each candidate sequence was evaluated by BLAST (NCBI) to confirm specificity and exclude off-target matches to the human genome or other sequences in the nonredundant database. Candidates with a GC content of 30–50%, which is generally associated with efficient RNAi activity [26,27,28], were selected.

A nonsilencing scrambled siRNA (scr siRNA) control, sharing base composition but lacking homology with EV71 sequences in NCBI, was also designed. All siRNAs used in this study were synthesized with 3′-dTdT overhangs on both strands and were labeled with a 5′ fluorescein amidite (FAM) at the 5′ end of the sense strand. The synthesized siRNAs (Bioneer, Daejeon, Republic of Korea) were reconstituted in ultrapure distilled water at 100 µM and stored at –80 °C until use.

### 4.4. Optimization of the Amount of siRNA

Individually designed siRNAs targeting the VP4, VP3, 2B, and 3A genes of EV71 were used as single treatments [11]. Cell viability assays were performed to assess potential cytotoxicity from siRNAs or the transfection reagent. A concentration of 25 nM was identified as optimal for single-siRNA application, showing minimal toxicity and stable expression for up to 72 h [11]. The same concentration was applied in the combination treatments to maintain a consistent total siRNA amount.

### 4.5. Transfection of siRNA

To evaluate the antiviral effects of the siRNAs, HeLa cells were transfected with either a single siRNA or a combination of multiple siRNAs. Scr siRNA-transfected and untreated cells served as controls, and mock-infected cells were included as negative controls. Transfection was carried out with Lipofectamine RNAiMAX 2000 (Invitrogen Life Technologies) according to the manufacturer’s instructions. Twenty-four hours post-transfection, cells were infected with EV71 at an MOI of 1 × 10^−3^ and incubated at 37 °C. The cells were collected at 24, 36, and 48 h post-infection, and viral replication was assessed by Western blot, immunofluorescence assay, and plaque assay.

### 4.6. Viral Escape from siRNA-Mediated Inhibition

The viral ability to evolve resistance to RNAi-mediated inhibition was investigated by serially passaging the virus in the presence of one or a combination of effective siRNAs. For the first passage (P1), cells were infected with the original stock virus (P0), and wells were monitored every 8 h (beginning at 24 h post infection) for the development of the cytopathic effect (CPE). When a particular treatment showed greater than 80% CPE, the culture media was harvested and stored at -80 °C. For subsequent passages (P2-P5), cells were transfected as described above and infected with identically treated cells from the previous passage. At the end of the experiment, all serially passaged viruses were titrated by plaque assay, and consensus sequencing was performed. To determine whether the mutations identified in viruses serially passaged in siRNA-treated cells conferred a resistant phenotype, the siRNA-treated cells were infected with the serially passaged viruses. The cells were transfected with a single siRNA, multiple siRNAs, or scr siRNA. Untreated and uninfected control wells were included in each experiment.

### 4.7. Plaque Assay

Viral titers were determined by plaque assay on Vero cells (CCL-81™, ATCC). Supernatants from EV71-infected cells were serially diluted 10-fold and applied to 90–95% confluent Vero monolayers. After 1 h of adsorption, the cells were washed with PBS and overlaid with 2× DMEM (Welgene, Gyeongsan, Republic of Korea) containing 2% agar (Lonza, Walkersville, MD, USA). The cultures were incubated at 37 °C for 72 h, fixed with 3.75% formaldehyde (Sigma-Aldrich, St. Louis, MO, USA), and stained with 1% crystal violet (Lugen Sci, Seoul, Republic of Korea). Plaques were counted, and viral titers were expressed as plaque-forming units/mL. Each assay was performed in triplicate.

### 4.8. Immunofluorescence

Cells were seeded on chamber slides one day before siRNA transfection and virus infection. After treatment, the cells were washed with PBS, fixed with 4% paraformaldehyde for 10 min, and permeabilized with 0.1% Triton X-100 (Sigma-Aldrich, St. Louis, MO, USA) in PBS containing 0.05% Tween^®^ 20 (PBST; Sigma-Aldrich) for 10 min. Blocking was performed with 1% bovine serum albumin (Sigma-Aldrich) in PBST for 30 min at 25 °C. The cells were then incubated overnight at 4 °C with an anti-EV71 primary antibody (Abcam, Cambridge, UK), followed by goat anti-mouse IgG (H + L) cross-adsorbed secondary antibody (Invitrogen Life Technologies, Carlsbad, CA, USA) for 1 h at 25 °C. Nuclei were counterstained with Hoechst 33,342 (Invitrogen Life Technologies) for 10 min at 25 °C. Images were acquired using an epifluorescence microscope (Carl Zeiss, Jena, Germany) and analyzed with Axio Vision software v4.0 (Carl Zeiss).

### 4.9. Western Blot Analysis

Cells were lysed on ice for 20 min with M-PER reagent (Invitrogen Life Technologies) supplemented with a protease/phosphatase inhibitor cocktail (Roche Diagnostics, Indianapolis, IN, USA). The lysates were collected by scraping and centrifuged at 14,000× *g* for 15 min at 4 °C. Protein concentrations were measured using the BCA Protein Assay Kit (Intron Biotechnology, Seongnam, Republic of Korea). Equal amounts of protein were separated by SDS–PAGE and transferred to PVDF membranes (Bio-Rad Laboratories, Hercules, CA, USA). The membranes were blocked for 1 h with 5% milk in TBS containing 0.05% Tween^®^ 20 (Bio-Rad Laboratories) and then incubated overnight at 4 °C with primary antibodies, followed by a 1 h incubation with secondary antibodies. Bands were visualized using an enhanced chemiluminescence system (GE Healthcare, Madison, WI, USA). The primary antibodies included anti-EV71 VP1 (GeneTex, Irvine, CA, USA), protein kinase R (PKR, dsRNA-activated protein kinase, Santa Cruz, CA, USA), p-PKR (Abcam, Cambridge, MA, USA), and β-actin (Santa Cruz Biotechnology). The secondary antibodies used in this study were anti-rabbit IgG, HRP-linked antibody (Cell Signaling Technology, Danvers, MA, USA), and anti-mouse IgG, HRP-linked antibody (Cell Signaling Technology). Specifically, protein samples were collected at 24 h post infection, a timepoint chosen to capture early viral gene expression and interferon responses.

### 4.10. Evaluation of Cytopathic Effects and Cell Viability

Cytopathic effects in HeLa cells following EV71 infection and siRNA treatment were monitored microscopically. Cell viability was evaluated both to determine the optimal siRNA concentration for antiviral assays and to verify that reductions in viral titers were due to siRNA activity rather than cell loss. Viability was measured using the EZ-Cytox Cell Viability Assay Kit (Daeillab Service, Seoul, Republic of Korea) according to the manufacturer’s instructions. The 48 h post-infection timepoint was selected because it corresponded to the stage at which most infected cells underwent virus-induced cell death.

### 4.11. Evaluation of Off-Target Effect (Activation of the Interferon Pathway)

To investigate whether the inhibition of EV71 by siRNA treatment was a nonspecific effect related to the interferon response, the phosphorylation pattern of PKR was examined as a dsRNA-activated protein kinase 2.

### 4.12. Statistical Analyses

All experiments were performed in three independent assays, each conducted in triplicate. Statistical analyses were carried out using Prism software (GraphPad Software, San Jose, CA, USA). Significance was assessed using Student’s t-test, two-way ANOVA with Bonferroni’s post-test, or one-way ANOVA with Bonferroni’s multiple comparison test. *p* < 0.05 was considered statistically significant.

## 5. Conclusions

This study supports the effectiveness of combination therapy using siRNAs targeting multiple genes to inhibit viral replication in vitro and prevent the emergence of resistant escape mutants. The findings highlight the benefits of RNAi mechanisms in the development of treatments targeting viral diseases, providing versatility and numerous targets even in small viral genomes. This approach offers promise for future antiviral treatments in an unexpected infectious disease outbreak caused by an emerging virus or for variant strains with unknown genomic sequences.

## Data Availability

The raw data supporting the conclusions of this article will be made available by the authors on request.
